# Storage Stability and In Vitro Bioaccessibility of Liposomal Betacyanins from Red Pitaya (*Hylocereus polyrhizus*)

**DOI:** 10.3390/molecules27041193

**Published:** 2022-02-10

**Authors:** Xian Lin, Bozhe Li, Jing Wen, Jijun Wu, Daobang Tang, Yuanshan Yu, Yujuan Xu, Baojun Xu

**Affiliations:** 1 Sericultural & Agri-Food Research Institute, Guangdong Academy of Agricultural Sciences/Key Laboratory of Functional Foods, Ministry of Agriculture and Rural Affairs/Guangdong Key Laboratory of Agricultural Products Processing, Guangzhou 510610, China; sannylam@126.com (X.L.); libozhe19@163.com (B.L.); wenjing@gdaas.cn (J.W.); guoshuwujijun@163.com (J.W.); tangdaobang@gdaas.cn (D.T.); yuyuanshan@gdaas.cn (Y.Y.); 2 Food Science and Technology Program, BNU-HKBU United International College, Zhuhai 519087, China

**Keywords:** red pitaya, betacyanin, liposome, storage stability, bioaccessibility

## Abstract

In order to address the poor stability of the betacyanins from red pitaya (*Hylocereus polyrhizus*, HP), which are considered as good sources of natural colorant, liposomal-encapsulation technique was applied in this study. Thin-layer dispersion method was employed to prepare HP betacyacnin liposomes (HPBL). The formulation parameters for HPBL were optimized, and the characteristics, stability, and release profile of HPBL in in vitro gastrointestinal systems were evaluated.Results showed that an HP betacyanin encapsulation efficiency of 93.43 ± 0.11% was obtained after formulation optimization. The HPBL exhibited a narrow size distribution of particle within a nanometer range and a strong electronegative ζ-potential. By liposomal encapsulation, storage stability of HP betacyanin was significantly enhanced in different storage temperatures. When the environmental pH ranged from 4.3–7.0, around 80% of HP betacyanins were preserved on Day 21 with the liposomal protection. The loss of 2,2′-Diphenyl-picrylhydrazyl (DPPH) scavenging activity and color deterioration of HPBL were developed in accordance with the degradation of HP betacyanins during storage. In in vitro gastrointestinal digestion study, with the protection of liposome, the retention rates of HP betacyanins in vitro were enhanced by 14% and 40% for gastric and intestinal digestion, respectively.This study suggested that liposomal encapsulation was an effective approach to stabilize HP betacyanins during storage and gastrointestinal digestion, but further investigations were needed to better optimize the liposomal formulation and understand the complex liposomal system.

## 1. Introduction

*Hylocereus* pecies, commonly known as pitaya or dragon fruit, belong to the order Caryophyllales. They originate from the American continent and are now cultivated on a large scale in South China, Malaysia, Vietnam, Thailand, and some other parts of the world. The fruit has rich sources of nutrients and abundant bioactive compounds including flavonoids, crude fiber, betalains, carotenes, and polyphenols [1]. *Hylocereus* *polyrhizus* (HP) is a kind of *Hylocereus* species with attractive red color in peel and flesh. The red color is endowed by water-soluble vacuolar pigments—betacyanins, which are immonium conjugates of betalamic acid with cyclo–DOPA [2]. Due to this, HP has been identified as a potential source of betacyanins that can be used as natural pigments in the food industry. Besides the appealing color, betacyanins are also considered as significant phytochemicals with potential health benefit effects for human beings. Numerous in vitro and ex vivo studies have confirmed that betacyanins possess antioxidant activity and have anti-tumor effects in animal models and in cancerous cell lines [3]. Therefore, the application of HP as a source of betacyanins pigments could have great potential on the natural colorant market.

However, the unstable physicochemical stability of betacyanins may cause significant loss of chromatic characteristics and bioactive potential during processing, storage, and digestion, limiting their applications as natural functional colorants. Previous studies have demonstrated that betacyanins were susceptible to environment factors. Exposure to light, oxygen, high water activity, metal ions, and high temperature could all lead to the degradation of betacyanins [4]. The instability of physicochemical property could also affect the bioavailability of betacyanins, which plays a vital role in the functioning of their biological properties. Krantz et al. compared the differences in excretion of betanin when the rats were given betanin from beetroot extract by intravenous injection or oral administration [5]. Results showed that when given by intravenous injection, betanin was almost completely recovered in the urine, but only 3% was recovered in the urine and 3% in the feces after 24 h when given orally. To date, a number of in vitro studies have evidenced the significant loss of betacyanins in stimulated GI digestion, limiting the bioaccessibility of betacyanins [3].

Liposome is self-assembled closed vesicle with a phospholipid bilayer structure, the particle size of which varies from 10 nm to several micrometers. Liposomes contain polar, non-polar, and amphiphilic regions within phospholipid bilayer compartment, which enable the encapsulation of both lipophilic and hydrophilic compounds [6]. The application of the liposomal system as a nano-carrier for the delivery of bioactive compounds has been considered as a potential tool to overcome series limitations associated with environmental-instability, poor solubility, gastrointestinal degradation, low bioavailability, uncontrolled-release, and unspecific delivery [7]. The stability of a liposome is crucial for the delivery of the entrapped compound. Preparation technique, operating parameters, and formulation parameters could all play a significant role in the liposomal preparation. To date, several bioactive compounds such as quercetin, vitamin C, curcumin, resveratrol, and capsaicin, have been successfully entrapped in liposomes with improved solubility, bioavailability, and biological activity [6]. 

Thus, in this study, in order to address the poor stability and bioavailability of HP betacyanins, liposomal-encapsulation technique was applied. Formulation parameters of HPBL by the thin-layer dispersion method were optimized based on the encapsulation efficiency. Moreover, characteristic, stability, and release profile of HPBL in invitro gastrointestinal systems were evaluated. The results could provide important information for the design of HPBL, aiming their application at the functional food industry.

## 2. Results and Discussions

### 2.1. Optimization of the Formulation Parameters for Preparation of HPBL

Model fitting. The values of responses of encapsulation efficiency (EE) of HP betacyanins at different experimental combinations are given in Table 1, which show considerable variations in the encapsulation efficiency of HP betacyanins under different thin-layer dispersion conditions. The multiple regression analysis was applied to the experiment data, and the response variable and the test variables can be expressed by the following second-order polynomial equation:

Y = 88.85 − 4.40X_1_ − 1.10X_2_ − 1.51X_3_ + 0.64X_1_X_2_ − 0.386X_1_X_3_ − 1.98X_2_X_3_ − 6.59X_1_^2^ + 1.03X_2_^2^ + 1.04X_3_^2^ (1)

where Y is the EE of HP betacyanins, and X_1_, X_2_, and X_3_ are the coded variables for HP extract concentration, lecithin-to-cholesterol ratio, and lecithin concentration, respectively.

The analysis of variance (ANOVA) is listed in Table 2 for the fitted quadratic polynomial model of EE of HP betacyanins. Results suggested that the model was significant (*p* < 0.05) with a non-significant lack of fit (*p* > 0.05), indicating it was adequate for predicting the EE of HP betacyanins under any combination of values of the variables. The variance of the quadratic regression model showed that the determination coefficient (R^2^) was 0.9536, implying that only 4.64% of the total variations were not explained by the model. An adjusted R^2^ of 0.8939 further confirmed the model was highly significant.

**Figure 1:**
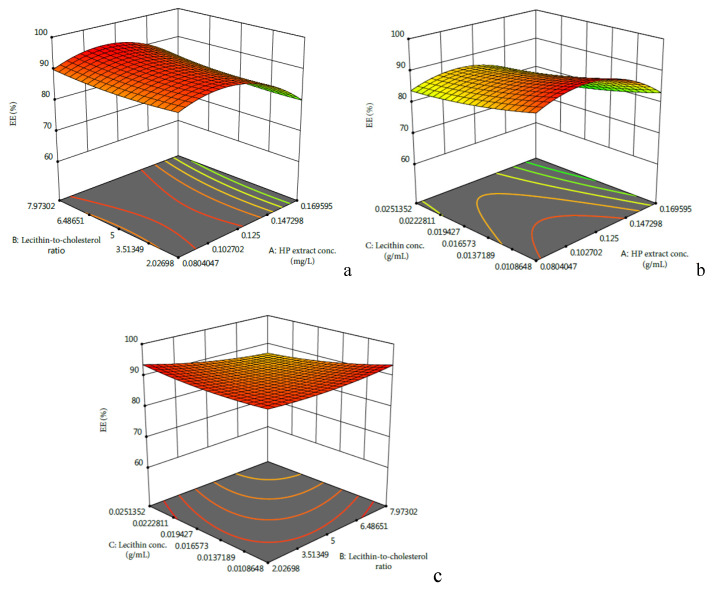
Response surface plots showing the effects of HP extract concentration, lecithin-to-cholesterol ratio, and lecithin concentration on the EE of HPBL: (**a**) interactive relationships between HP extract concentration and lecithin-to-cholesterol ratio; (**b**) interactive relationships between HPextract concentration and lecithin concentration; (**c**) interactive relationships betweenlecithin-to-cholesterol ratio and lecithin concentration.

As shown in Table 2, independent variable A and quadratic term A^2^ significantly affected the EE of HP betacyanins. From Figure 1a,b, it was seen that the effects of HP extract concentration on the EE of HP betacyanins presented a peak in the EE of HP betacyanins when the HP extract concentration ranged from 0.050 to 0.200 g/mL. The results suggested that at lower and higher level of extract concentration, EE was positively and negatively related with the extract concentration, respectively. Similarly, Guldiken et al. found that when the concentration of anthocyanin-rich black carrot extract increased from 10% to 40%, the EE of phenolics decreased accordingly [8]. Zhao et al. found an increase of anthocyanin concentration from 0.1% to 0.5% led to a significant increase *(p* < 0.05) in the EE of anthocyanins [9]. 

Figure 1c indicated that the EE of HPBL increased with the decrease of B or C. However, as Table 2 suggested, the effects of factor B and C, as well as their interactive relationships, were not significant (*p* > 0.05). Thus, in order to maximize the lecithin-to-cholesterol ration and minimize the lecithin concentration, the optimized parameters were determined to be an HP extract concentration of 0.104 g/mL, a lecithin-to-cholesterol ratio of 7.9:1, and a lecithin concentration of 0.011 g/mL, which was predicted to have an EE of 93.30%.

Validation ofthe model. To validate the accuracy of the model equation, a further experiment was carried out with the optimal condition. Results showed the practical EE was 93.43 ± 0.11%, which matched well with the predicted value. Thus, it was confirmed that the regression model was accurate and adequate for the prediction of encapsulation efficiency of HP betacyanins.

### 2.2. Characterization of the HPBL

**Figure 2:**
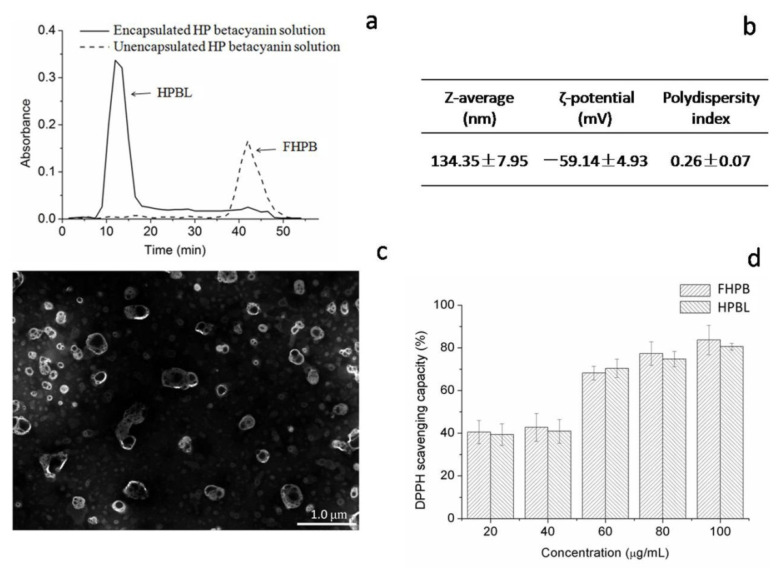
Physicochemical characteristics of HPBL: (**a**) Absorbance of encapsulated HP betacyanin solution and unencapsulated HP betacyanin (FHPB) solution with a wavelength of 538 nm as separated by Sephadex G-50; (**b**) size(z-average), ζ-potential, and polydispersity of HPBL;(**c**) transmission electron microscopy observation of HPBL; (**d**) DPPH free radical scavenging rate.

The performance of liposomes is strongly related to their characteristics. Liposomes can be characterized in terms of ζ-potential, particle size, and polydispersity index [8]. Results of characterization of HPBL are shown in Figure 2. As seen in Figure 2a, results of Sephadex^®^ G50 filtration displayed the separation of HPBL and unencapsulated HP betacyanins (FHPB), as the peak of HPBL occurred only in the HPBL dispersion group at a wavelength of 538 nm, which was the wavelength of the absorption maximum of betacyanins.

Particle size is an important factor of liposomes for the oral delivery of bioactive compounds, as particle size determined the absorption efficiency in the gastrointestinal tract, biodistribution, and pharmacokinetic profiles [9]. Andar et al. investigated the cellular uptake of liposomes with mean diameters ranging from 40.6 nm to 276.6 nm in Caco-2 cells and found the uptake of liposome was markedly negatively correlated with its size [10]. It was also reported that as the size of liposome decreased from 400 to 40 nm, a longer blood circulation was observed. In addition, the prolonged blood circulation tended to enhance the accumulation of entrapped ingredients in targeted sites. Based on the collected data, Nagayasu et al. concluded that the maximum tumor accumulation of liposomes was around 100 nm in diameter [11]. In the current study, as Figure 2b shows, the z-average of HPBL was 134.35 ± 7.95 nm, which is in optimal size range and could enhance the bioavailability by oral delivery.

The ζ-potential is regarded as an indicator of particle stability in the colloidal system. With a high ζ-potential, normally above 30 mV or below –30 mV, particles will repel each other and decrease the tendency of aggregation and enhance the dispersibility in the aqueous media [12]. As seen in Figure 2b, the ζ-potential of HPBL presented a strong electronegative value, which was below –30 mV, suggesting the HPBL was physically stable. Compared to the ζ-potential of betanin-loaded liposomes of −19.05 ± 1.65 mV, the HPBL’s ζ-potential was greatly increased, which could be attributed to the more negative charges offered by the HP betacyanins [13].

Another source of information on the quality of a liposome formulation is the PDI. The higher PDI values of the liposomes indicated a broader size distribution or the development of a bimodal particle size distribution. PDI < 0.3 is considered as an ideal and narrow size distribution of particle [6]. Figure 2b displays that the PDI of HPBL was in the range of 0.2 to 0.3, indicating a low polydispersity. This value was in accordance with the results of Da Silva Malheiros et al. and Marín et al., as liposomes prepared from biological materials were expected in this range [12,14].

The morphology of HPBL was observed by transmission electron microscopy (TEM) as shown in Figure 2c. It was confirmed that the sizes of HPBLs were mainly around 134.35 nm, which matched the results of the particle size. Moreover, the morphology of HPBL appeared to be heterogeneous vesicles, which was similar with those liposomes prepared in the studies of Amjadi et al. and Zhao & Temelli, as the thin film hydration method led to the production of liposomes with unilamellar and multilamellar structures [13,15]. The complex composition of extracted HP betacyanins solution and the capacity of liposomes to encapsulate compounds with different polarities could also be explained for the irregular shape of HPBL instead of the spherical shape. Owning bothwater and lipid phase, liposomes could encapsulate hydrophobic, hydrophilic, or amphiphilic compounds. For hydrophilic compounds like betacyanins, they could be entrapped in the inner aqueous phase or adhered on the surfaces of the liposomes. For hydrophobic compounds like some flavonoids, they could be embedded between the lipid bilayers of liposomes [6].

Betacyanins, which are similar with phenolic compounds in terms of structure and function, were demonstrated to be excellent antioxidant phytochemicals [16]. Evidences showed that betacyanins could protect against oxidative stress-related diseases, owing to it strong antioxidant capacity. The antioxidant capacity of betacyanins was reported to be due to their high radical scavenging activity. The radical scavenging activity of betacyanin functions by being oxidized by free radicals. Once oxidized, the structure of betacyanins will be broken down into thecore structure betalamic acid and other small molecules [17]. There are various methods to evaluate the radical scavenging activity. Among them, the DPPH assay is a frequently used method as it can rapidly determine the radical scavenging activity.The DPPH scavenging activity of the betacyanins could be determined by the numbers of hydroxyl/imino groups, the positions of hydroxyl groups, and glycosylation of aglycones in the betacyanin molecules [18].Thus, different betacyanins have different levels of free radical scavenging activity due to the distinction of the structure. For example, betanidin with a catechol group possessed greater free radical scavenging activity than those of the glycoside of betacyanins [18]. Figure 2d displays the DPPH scavenging activity of FHPB and HPBL between betacyanin concentrations of 20 and 100 μg/mL. Results showed that no significant difference was observed between FHPB and HPBL, suggesting that liposomal encapsulation had no impact on the antioxidant capacity of HP betacyanins. Similarly, Malheiros found liposomal encapsulation did not affect the biological activity of the loaded bioactive compounds [14].

### 2.3. Storage Stability of HPBL

#### 2.3.1. Temperature

**Figure 3:**
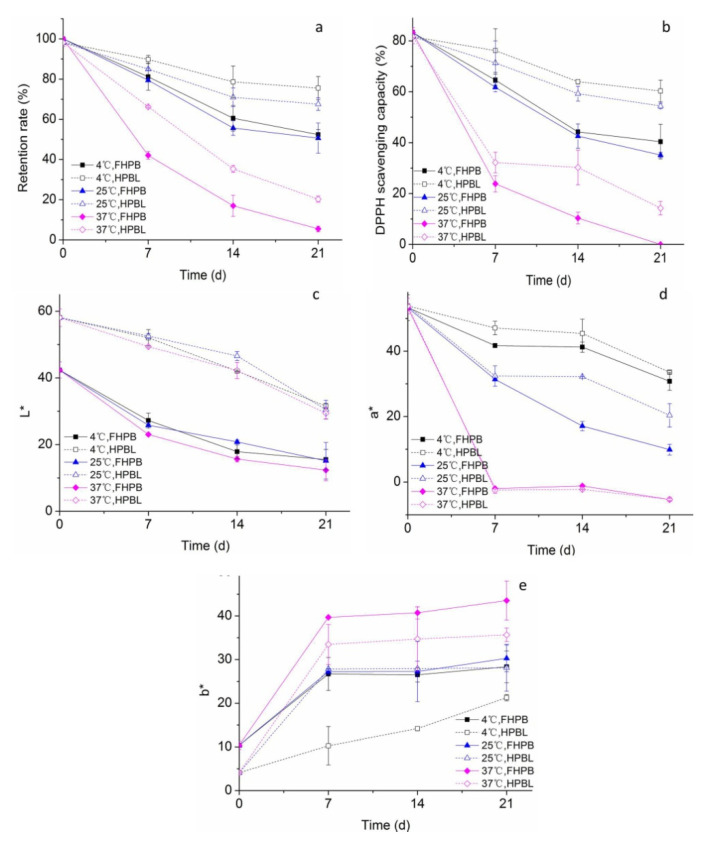
Stability of HPBL under different storage temperatures: (**a**) Retention rate; (**b**) DPPH scavenging activity; (**c**) L* value, which represents the color of lightness; (**d**) a* value, which represents the color redness when the value is positive; and (**e**) b* value, which represents the color of yellowness when the value is positive.

The stability of HPBL in storage temperature conditions of 4 °C, 25 °C, and 37 °C is shown in Figure 3. As Figure 3a displays, the betacyanin retention rate decreased with the increase of storage temperature. Especially, when the storage temperature raised from 25 °C to 37 °C, the retention rate of FHPB dropped by 89% on Day 21. By comparison with FHPB, betacyanin retention in HPBL was enhanced in all storage temperature conditions during a storage period of 21 days, indicating the protective effects of liposomal encapsulation. The retention rates of HPBL storing at 4 °C, 25 °C and 37 °C reached 75.54%, 67.57%, and 20.28% on Day 21, which were improved by 44%, 33% and 271%, respectively. However, when stored at a high temperature of 37 °C, even with liposomal encapsulation, the betacyanin retention rate was only 11.06% by the end of the storage, showing the severe heat-sensitivity of HPBL. Consistent with our current results, Amjadi et al. successfully improved the stability of betanin in gummy candies by liposomal encapsulation of betanins and prevented the betanin from the hydrolysis and oxidation conditions [18]. Liu et al. found that, in the temperature variation range of 20–90 °C, the stability of curcumin was significantly improved during storage with the protection of liposome [19]. However, the retention rate of liposomal betacyanins still decreased significantly.

Temperature is a crucial influencing factor for both betacyanin and liposome stability. Generally, the liposomal particles have a high tendency to degrade, flocculate, fusion, or aggregate, leading to the leakage of entrapped bioactive compounds during storage or after administration [19]. Release of the encapsulated compound in liposome depends greatly on the characteristic of the compound. For hydrophobic compounds, their diffusion from the bilayer core to the aqueous solution could be inhibited due to the interactions, such as van der Waals’ interactions, hydrophobic interactions, and hydrogen bonds. However, the release of a hydrophilic compound could probably happen by permeating across the liposomal membrane, to some extent which depends on the structure of the liposome and the properties of the encapsulated compound [20]. Maherani et al. revealed that the hydrophilic compound calcein could permeate across the liposomal membrane without membrane disruption [21]. As hydrophilic compounds, betacyanins might be encapsulated in the water phase of the liposome, embedded in the core of the liposome, or just adhering to the surface of liposome by electrostatic repulsive force [18]. Thus, betacyanins entrapped in the core of liposomes could be released by penetrating and crossing the liposomal membranes, and those adhering on the surfaces of liposomes might be isolated and released, leading to the gradual exposure of betacyanins to the environment during storage. With the increase of environmental temperature, the hydrophobic interactions and van der Waals forces in the liposomal bilayers became weaker and the permeability of bilayers increased, thus facilitating the leakage of the encapsulated compounds [6]. In addition, betacyanins are heat-sensitive [22]. Therefore, the increase of environmental temperature accelerated the degradation of liposomal betacyanins.

As Figure 3b displays, the variations of DPPH scavenging capacity decreased in a similar manner with the betacyanin retention rate, suggesting that betacyanin concentration could be the major factor responsible for the antioxidant capacity of HPBL dispersion. By liposomal encapsulation, the DPPH scavenging capacity of HP betacyanin was effectively maintained during the storage. Similar to this study, Gandía-Herrero et al.found that, with encapsulation by matrixes of chitosan and maltodextrin, the stability and antioxidant capacities of betacyanins were effectively retained [23].

Color is the most important feature for betacyanins, when it is developed to be a natural colorant. Figure 3c–e presents the color variation of the samples. As shown in Figure 3c, HPBL samples showed an obvious higher whiteness than FHPB samples. Similarly, Amjadi et al. also observed that the L* value was elevated in liposomal betanin, as the whiter appearance was conferred by lecithin [18]. With the increase of storage time, the whiteness of both HPBL and FHPB decreased gradually in all groups. As Figure 3d showed, the variations of a* value also had high consistency with the betacyanin retention rate. Figure 3e shows that b* values of all samples increased during the storage period, suggesting the increase of the yellowness of the samples. This may be attributed to the degradation of betacyanins. The yellowness also increased with the increase of temperature. Storing at 37 °C, the yellowness of FHPB was increased by 54% as compared to those at 4 °C on Day21. With the protection of liposome, the shift to yellowness was significantly restrained. Consistently, treating the betacyanin-rich red beet juice with 85 °C for 8 h, a shift of the red-purple hues of red beet juice to yellow-orange ones was also found [18].

#### 2.3.2. PH

**Figure 4:**
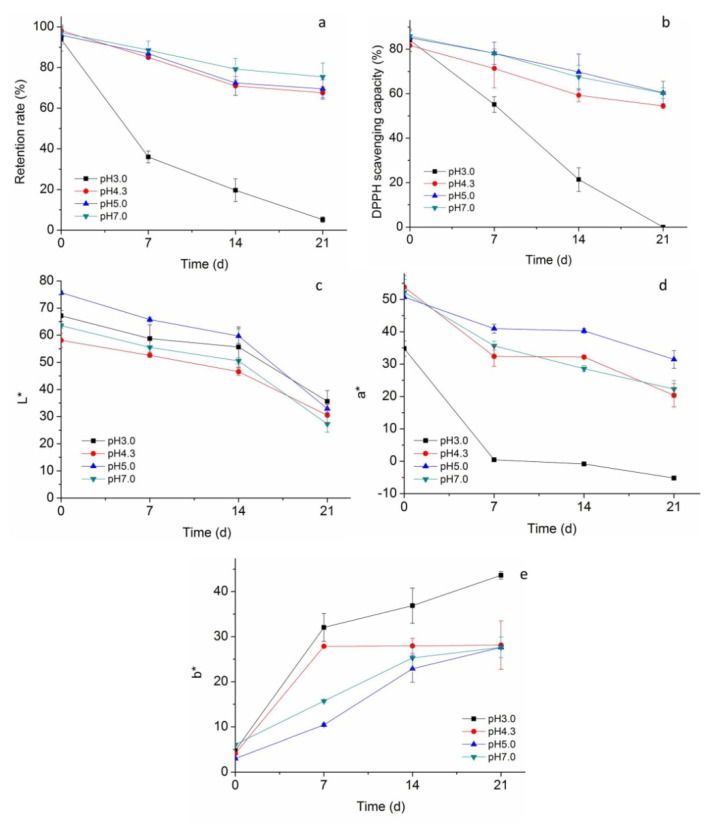
Stability of HPBL at different pH: (**a**) Retention rate; (**b**) DPPH scavenging activity; (**c**) L* value, which represents the color of lightness; (**d**) a* value, which represents the color rednesswhen the value is positive; (**e**) b* value, which represents the color of yellowness when the value is positive.

Previous studies reported that betacyanins were stable at a pH range of pH 3 to 7 [24]. When the pH was over 7, the color of betacyanins was altered to be yellowish-brown [25]. Thus, changes of stability of HPBL were evaluated in pH between pH 3–7. As Figure 4a shows, the variations of HPBL in pH conditions from 4.3–7.0 had similar patterns, which declined gently during storage and around 80% of betacyanins were preserved on Day 21. It is noted that the highest betacyanin retention rate during the storage was observed at pH 7.0. However, in conditions of pH 3.0, betacyanin retention rate of HPBL dropped markedly during storage, with over half of betacyanins degraded within 7 days.

Similar to the factor of temperature, the stability of the liposomes and encapsulated betacyaninsin different pH conditions could both affect the retention rate of liposomal betacyanins. Low pH condition could alter the surface charges of the liposome and lead to the aggregation of the liposomes when the electrostatic repulsive force is not big enough to sustain the stabilization of the dispersion [6]. However, the major reasons for the substantial reduction of liposomal acidic condition could be due to the degradation of the betacyanins released from the liposome. Similar to our results, Gandía-Herrero et al. found that the stability of betaxanthin was more stable at pH 6 and 7 than at the pH values of 4, 5, and 8 [26]. Montes-Lora et al. found that betacyanins in the *Ullucus tuberosus* and *Opuntia dillenii* were more suitable in low-acidic foods (pH 5 and 6) than in pH 4 condition [27]. The reasons for different stable ranges of pH may be attributed to the different environmental conditions as well as the different chemical structures of different betacyanins [28].

As Figure 4b shows, the variation of DPPH scavenging activity was in high consistency with the retention rate. In pH conditions of 4.3–7.0, the DPPH scavenging activity was well maintained. Notably, the DPPH scavenging activity of HPBL was reduced by only 29% and 30% on Day 21 in pH 5.0 and 7.0 conditions, respectively. However, the DPPH scavenging activity of HPBL in pH 3.0 plummeted from 88.07% to 0 during the 21 days storage. In accordance with our results, Gliszczynska-Swiglo et al. found that the free radical scavenging activity of betanin was pH-dependent, which was more active than some anthocyanins when the pH value was over 4 [29]. The pH-dependent increase of free radical scavenging activity of betacyanins could be attributed to the formation of its different deprotonated forms. With the increase of pH, betacyanins became better electron donators, thus leading to the increase in its free radical scavenging activity.Gandía-Herrero et al. investigated the free radical scavenging capacities of the structural unit of betalians–betalamic acid and also found the pH-dependence [30]. In addition, it was reported that betalamic acid displayed a notable increase in the radical scavenging capacity when the pH value was above pH 5.5. As for the color, Figure 4c shows the L* value of HPBL was susceptible to the pH conditions. The L* value changed the moment the pH was adjusted to a different value, and HPBL at pH 5.0 and pH 4.3 displayed the highest and the lowest whiteness, respectively. The whiteness of all groups decreased during the storage, especially with a higher rate from Day 14 to Day 21. Figure 4d showed that the variation of a* value (redness) was most like that of the betacyanins retention rate. Notably, a* value of HPBL in pH 3.0 decreased steeply and even reached minus value on Day 21, indicating the diminishing of redness and appearance of greenness. For the b* value, as Figure 4e displays, little difference was observed on Day 0. However, after 7 days of storage, the b* values of HPBL in pH 3.0 and 4.3 conditions were significantly higher than those in pH 5.0 and 7.0, indicating a more acidic environment led to the increase of yellowness. With a further increase of storage time, differences in the b* value of HPBL in pH 4.3, 5.0, and 7.0 conditions gradually uniformized, but that of the HPBL in pH 3.0 continuously went up and reached 43.62 on Day 21. The reason for severe discoloration of HPBL in pH 3.0 environment may be related to the unstable property of HP betacyanins and the instability of liposomes in this condition. As betacyanins are susceptible to hydrolysis at acidic pH, similarly, Rodríguez-Sánchez et al. found that betaxanthins in *Stenocereus* *pruinosus* were most stable at pH 6.6 [31]. With the decrease of environmental pH value, the betaxanthin retention decreased accordingly. For liposomes, Liu found that in a low pH environment, the liposomes aggregated as the electrostatic repulsive force reduced [32]. Overall, the color of HPBL was highly related with the betacyanin retention rate andwas more stable in a low acidity environment. The degradation of betacyanins led to a gradual reduction of redness and the appearance of a brown shade. 

### 2.4. In Vitro Gastrointestinal Stability of HPBL

**Figure 5:**
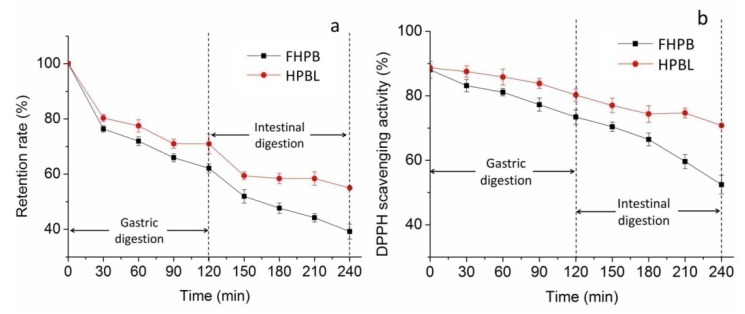
Stability of HPBL during in vitro gastrointestinal digestion: (**a**) Retention rate of HPBL during in vitro GI digestion; (**b**) variation of DPPH scavenging activity of HPBL during in vitro GI digestion.

The stability of the HPBL during the digestion process was evaluated under simulating digestion conditions of the stomach and the intestine. Betacyanin retention rate of HPBL during in vitro gastric and intestinal digestion is shown in Figure 5a. As it is shown, betacyanin retention rates of both FHPB and HPBL decreased gradually with the increase of time in the stimulated gastric digestion process. After 120 min of gastric digestion, the betacyanin retention rate of HPBL reached 70.96% and increased by 14% as compared to that of the FHPB. Betacyanin retention rate exhibited similar variation in intestinal digestion process. The betacyanin retention rate of FHPB kept going down with the increase of digestion time and finally reached 39.20%, indicating the instability of FHPB in intestinal digestion conditions. However, it is noted that after a significant decrease in the first 30 min of intestinal digestion, the betacyanin retention rate of HPBL maintained well at around 58% in the rest time. At the end of gastrointestinal digestion, the betacyanin retention rate of HPBL was increased by 40% as compared to that of the FHPB.

Figure 5b displays the variations of DPPH scavenging activity of HPBL in stimulated gastrointestinal process. As it is shown, the DPPH scavenging activity of FHPB and HPBL underwent a similar changing pattern with the betacyanin retention rate, suggesting that the antioxidant activity of HPBL was highly correlated with the betacyanin concentration. By protection of the liposome, the DPPH scavenging activity of HPBL was improved by 9% and 35% at the end of gastric and intestinal digestion process, respectively. 

Previous studies have demonstrated the poor bioavailability of betacyanins by oral administration [16]. In this study, FHPB declined significantly in both the gastric and intestinal digestion process, due to the high acidic environment in the gastric digestion process and the effects of digestive enzymes and bile salts in the intestinal environment. Similar to our results, a previous study found about 75% and 35% betanin was metabolized in the stomach and small intestine in vitro, respectively, suggesting that the degradation of betanin in the gastrointestinal tract contributed greatly to its low bioavailability [5]. Tesoriere et al. found a loss of about 50% in betanin in in vitro oral, gastric, and small intestinal digestion [33]. However, in contrast to our results, Tesoriere et al. also found that betanin remained stable in the gastric digestion process [33]. Montiel-Sánchez et al. found a relative higher stability of betacyanins in cactus berry fruit in the gastric environment than in the intestinal environment [34]. The differences could be explained by the different compositions of betacyanins and the effects of the food matrix [16]. 

By protection of liposomes, the bioaccessibility of betacyanin was significantly sustained. Consistent with our results, liposomes have been demonstrated to be capable of protecting the targeting ingredients in the gastrointestinal system. Altin et al. encapsulated cocoa hull waste phenolic extract by chitosan-coated liposomes, and found the invitro bioaccessibility was two-fold higher in terms of total phenolic content, total flavonoid content, and total antioxidant capacity [35]. Seguin et al. also found that liposomal fisetin allowed a 47-fold increase in relative bioavailability compared to free fisetin and markedly improved its anticancer efficacy in mice [36]. 

There is no doubt that the retention of the encapsulated compound in liposomes are closely related with its release behavior, which determines its site of action in the gastrointestinal tract and bioaccessibility. The release behavior of liposomes in the digestion system mainly depends on the environmental parameters, characteristics of the encapsulated compound, and the properties of liposomes. In the aspect of environmental factors, digestive enzymes, pH, the composition and concentration of entrapped compounds, digestion time, and the composition of food matrices are all accounted [32]. 

Previous studies verified that the structure of liposome remained almost intact, as the gastric lipase had no activity on phospholipids [37]. However, low pH in the gastric environment could lead to the aggregation of liposomes induced by the reduction in electrostatic repulsive force among liposomes [32]. In this study, about 30% of loss was observed in betacyanins retention in HPBL in the gastric digestion process. Similarly, evidence showed that the release of lutein and *β*-carotene was found to be slow, but the release of lycopene and canthaxanthin was fast in the gastrointestinal system [20]. The reason for the degradation of the entrapped compound without disruption of the liposome may be related to the characteristics of the entrapped compound in the liposome. As mentioned above, entrapped betacyanins could be released from the liposome by penetrating and crossing the liposomal membrane. In addition, some of the betacyanins were just adhered on the surfaces of liposomes. Such betacyanins were exposed to the gastric juice and degraded in the acidic environment.

For the intestinal environment, Liu et al. reported the structure of liposome was greatly damaged in the intestinal digestion process [32]. The pancreatic enzymes including pancreatic lipase, phospholipase A2, and cholesterol esterase, led to the break of the lipid bilayer structure of liposome, and the bile salts further destroyed the phospholipid bilayer [32]. In addition, by induction of bile salts, liposomes could be reconstructed to micelle, which had such an extremely fine particle size that it could permeate and absorb between cells through membrane internalization and lymphatic transportation [32]. Due to the above reasons, the destruction of the liposomes in the intestinal environment could promote the release of betacyanins, making the betacyanins available for further intestinal absorption. Meanwhile, the destruction of the liposomes also led the degradation of some released betacyanins. Our results demonstrated that the betacyanin retention rate remained stable from 30 to120 min of intestinal digestion. It could be deduced that the betacyanins might be protected in phospholipid micelles, which were the digestive products of liposomes by bile salts with fine particle size [6].

Another important factor affecting the release behavior of liposomal ingredients is the properties of liposomes. Liposomal size, thickness, and number of layers of the membrane, as well as modification of liposomal membranes could all affect the release behavior of the encapsulated ingredients [6]. As this was an initial attempt to prepare HPBL, further studies would be conducted to improve the properties of liposomes so as to better control the release of HPBL in gastric digestion process and enable more betacyanins to reach the intestine in active forms.

## 3. Materials and Methods

### 3.1. Chemicals and Reagents

Red pitaya fruits were purchased from a local market in Guangzhou. Soy lecithin and cholesterol were of analytical grade and purchased from Yuanye Biotech Co., Ltd. (Shanghai, China). Sephadex G50 was obtained from Yika Biotech Co., Ltd. (Shanghai, China). DPPH was obtained from Sigma Chemical Company (St. Louis, MO, USA). Pepsin (10,000 u/mg), pancreatin (250 USPu/mg), and bile salts from pig were purchased from Yuanye Biotech Co., Ltd. (Shanghai, China). All other reagents used were analytical grade.

### 3.2. Preparation of HPBL

#### 3.2.1. Extraction of Betacyanins

The extraction of betacyanins was conducted according to Song’s methods with some modifications [38]. Four hundred grams of HP pulps were homogenized with 800 mL of cold acidic ethanol solution (80% ethanol and 0.1% acetic acid). After storing at 4 °C for 24 h, the mixture was centrifugated (4 °C, 5000 rpm, 10 min) and the supernatant was collected. Subsequently, the supernatant was concentrated in vacuum at 38 °C to remove the ethanol, and the betacyanins extracts were obtained.

#### 3.2.2. Quantification of Betacyanin Content

Determination of betacyanin contents was performed by the spectrophotometric method, according to Wu’s method with some modifications [39].The betacyanin content was assessed as betanin equivalents by the following equation:

(2) $$\mathrm{Concentration}\;\mathrm{of}\;\mathrm{betacyanins}\;\left(\mathrm{mg}/L\right)=\frac{A_{538}\times \mathrm{MW}\times \mathrm{DF}\times 1000}{\varepsilon \times L}$$

where A_538_ is the absorbance value at 538 nm; MW is the molecular weight of betanin (550 g/mol); DF is the dilution factor; ε is the molar extinction coefficient of betanin (61,600 L/(mol·cm)); and L is the path length of the cuvette. All determinations were performed in triplicate.

#### 3.2.3. Preparation of Liposomes

The liposome was prepared by the thin-layer dispersion method according to Amjadi’s methods with some modifications [13]. Certain amounts of lecithin and cholesterol were dissolved in ethanol. The ethanol was then removed by rotary evaporator at 50 °C under vacuum (EYELA N-1000, Tokyo Rikakikai Co., Ltd., Tokyo, Japan), and a thin layer was formed on the wall of the round bottom flask. The thin layer was then dissolved by adding 50 mL of distilled water and transferred to a 100 mL volumetric flask. Subsequently, the solution was diluted to 100 mL by adding certain amounts of HP betacyanin extracts. Then, the liposomal suspension was homogenized for 10 min twice in an ice bath. Finally, the liposomal dispersions were sonicated at an intensity of 600 W using an ultrasonic cleaner for 1 min eight times in an ice bath, and the HPBL solution was prepared. 

#### 3.2.4. Separation of HPBL and FHPB

The HPBL solution was extracted with ethyl acetate. Then, the aqueous layer was collected and applied to gel filtration chromatography of 1 × 20 cm column packed with pre-swollen Sephadex^®^ G50. Subsequently, samples were eluted by deionized water at a flow rate of 1 mL/min. The fractions were collected and detected by the spectrophotometer at 538 nm.

#### 3.2.5. Determination of EE

Concentration of FHPB was determined, and the encapsulation efficiency (EE) was calculated according to the following equation. All determinations were performed in triplicate.

(3) $$\mathrm{EE}\;\left(\%\right)=\frac{\mathrm{Total}\;\mathrm{betacyanin}\;\mathrm{content}-\mathrm{FHPB}\;\mathrm{content}}{\mathrm{Total}\;\mathrm{betacyanin}\;\mathrm{content}}\times 100$$

### 3.3. Optimization of the Formulation Parameters for Preparation of HPBL

Central composite design was employed to optimize formulation parameters for encapsulation as shown in Table 1. The software Design Expert was employed for experimental design, data analysis, and model building. Three variables used in this study were HP extract concentration (X_1_), lecithin-to-cholesterol ratio (X_2_), and lecithin concentration (X_3_), respectively, with three levels for each variable, while the dependent variable was the EE of HP betacyanins. The triplicates were performed at all design points in randomized order. Experimental data were fitted to a quadratic polynomial model and regression coefficients were obtained. The non-linear computer-generated quadratic model used in the response surface was as follows:

(4) $$Y_{0}=\beta_{0}+\sum_{j=1}^{k}\beta_{j}X_{j}+\sum_{j=1}^{k}\beta_{jj}X_{j}^{2}+\sum \sum_{i≺j}\beta_{ji}X_{i}X_{j}$$

where Y_0_ is the measured response associated with each factor lever combination; and β_0_, β_j_, β_jj_, and β_ji_ are the regression coefficients for intercept, linearity, square, and interaction; while X_i_ and X_j_ are the independent coded variables, respectively.

### 3.4. Analysis of Properties of HPBL

#### 3.4.1. Determination of ζ-Potential, Particle Size, and Polydispersity Index

ζ-Potential, particle size, and polydispersity index (PDI) of HPBL were determined with a Nano particle analyzer (SZ-100Z, HORIBA, Ltd., Tokyo, Japan). All measurements were performed at 25 °C. Each measurement of particle size and PDI was performed with a scattering angle of 90°. All determinations were performed in triplicate.

#### 3.4.2. Morphology Observation

Morphology of HPBL was observed by TEM. A drop of liposome dispersion was placed on a 200 mesh copper grid with formvar/carbon film, adhered for 3 min, and blotted dry. Then, samples were stained with phosphotungstic acid at 1% (*w*/*v*) for 3 min. After natural drying, specimens were observed using a TEM (HT7700, Hitachi Co., Ltd., Tokyo, Japan) at an accelerating voltage of 80.0 kV.

#### 3.4.3. Determination of DPPH Radical Scavenging Activity 

DPPH radical scavenging activity of betacyanins was evaluated according to Brand-Williams’s method with some modifications [40]. Two milliliters of the diluted samples were mixed with 2 mL DPPH solution (0.14 mmol/L) properly and kept in the dark at ambient temperature for 30 min. Then, the absorbance of the mixture was measured at 517 nm. The control was performed using distilled water to take the place of the sample. The blank was carried out using ethanol. The DPPH radical scavenging activity was determined through the following formula:

(5) $$\mathrm{DPPH}\;\mathrm{radical}\;\mathrm{scavenging}\;\mathrm{activity}=\frac{A_{\mathrm{control}}-A_{\mathrm{sample}}}{A_{\mathrm{control}}}\times 100\%$$

where $A_{\mathrm{control}}$ is the absorbance value of the control; $A_{\mathrm{sample}}$ is the absorbance value of the sample. All determinations were performed in triplicate.

#### 3.4.4. Evaluation of Color Value

The color of the sample was measured using a colorimeter (Ultrascan VIS, Hunter Associates Laboratory, Reston, VA, USA) fitted with a 25 mm diameter aperture. The colorimeter was calibrated using the black and white tiles provided. Evaluation of the color of HPBL suspension was expressed by L*, a*, and b* values, which represented the whiteness, the redness when positive, and the yellowness when positive, respectively. 

### 3.5. Storage Stability Evaluation of HPBL

#### 3.5.1. Temperature Stability Evaluation

To determine the temperature stability of HPBL, HPBL dispersions were stored at 4 °C (refrigerated condition), 25 °C (ambient condition), and 37 °C (accelerated condition) for 21 days. Samples were collected at an interval of 7 days, and color values were determined. Before analyzing the betacyanin retention rate and DPPH radical scavenging activity, samples were diluted with methanol (1:10 *v*/*v*) and sonicated at an intensity of 600 W for 10 min to break the liposomes. All determinations were performed in triplicate.

#### 3.5.2. PH Stability Evaluation

To determine the pH stability of HPBL, pH value of HPBL dispersion was adjusted to pH 3, 5, and 7, respectively. Coupled with the original pH 4.3 of the HPBL dispersion, the stabilities of HPBL in the four different pH conditions were evaluated at 25 °C for a storage of 21 days. Samples were collected at an interval of 7 days, and color values were determined. Before analyzing the betacyanin retention rate, and DPPH radical scavenging activity, samples were diluted with methanol (1:10 *v*/*v*) and sonicated at an intensity of 600 W for 10 min to break the liposomes. All determinations were performed in triplicate.

### 3.6. In Vitro Gastrointestinal Stability Evaluation of HPBL

An in vitro simulated gastrointestinal tract (GIT) model was applied according to Tai’s method with some modifications [41]. The GIT model included gastric phase and small intestine phase. For the stimulated gastric juice (SGJ), 800 mL of distilled water were mixed with 16.4 mL of 5 M HCl. Then the pH was adjusted to 1.2 by dropwise addition of 1 M HCl. After sterilization, the mixture was added with 10 g of pepsin. For the stimulated small intestinal juice (SIJ), 6.8 g of K_2_HPO_4_ were dissolved in 500 mL of distilled water, and the pH was adjusted to 6.8 by dropwise addition of 0.1 M NaOH. After sterilization, the mixture was added with 2 g of bile salts and 10 g of pancreatin.

All simulated digestive juices and liposomes were preheated at 37 °C, and the whole stimulating digestive process was conducted at a speed of 95 rpm in 37 °C water bath. Firstly, for stimulating gastric digestion, HPBL solution and SGF were mixed 1:1 by volume and stirring for 2 h. Samples were collected at an interval of 30 min. Then, the pH of the gastric digestive solution was adjusted to 6.8 and mixed with SIF by a volume ratio of 1:1. During the 2 h of stimulated small intestinal digestion, samples were also collected at an interval of 30 min. For calculation of the betacyanin retention rate and DPPH scavenging capacity, digestive samples were diluted with methanol (1:10 *v*/*v*) and sonicated at an intensity of 600 W for 10 min to break the liposomes. The betacyanin retention rate was calculated according to the following equation:

(6) $$\mathrm{Retention}\;\mathrm{rate}=\frac{\mathrm{Final}\;\mathrm{total}\;\mathrm{betacyanin}\;\mathrm{content}}{\mathrm{Initial}\;\mathrm{total}\;\mathrm{betacyanin}\;\mathrm{content}}\times 100\%$$

### 3.7. Statistical Analysis

Each experiment was performed in triplicate, and three samples from each treatment at each sampling day were analyzed. Results were expressed as mean ± standard deviation (SD). Comparisons of means were determined by ANOVA (analysis of variance) using SPSS 19 (SPSS Inc., Chicago, IL, USA) with significance set at *p* < 0.05.

## 4. Conclusions

In this study, liposomes technology was applied for encapsulation of HP betacyanins. The formulation parameters of HP extract concentration, lecithin-to-cholesterol ratio, and lecithin concentration were optimized to achieve an HP betacyanin EE of 93.43 ± 0.11%. The HPBL was characterized to be in nanometer range, physically stable, and have no impact on the antioxidant capacity of HP betacyanins. By liposomal encapsulation, HP betacyanin retention was demonstrated to be greatly enhanced in different storage temperatures (4 °C, 25 °C and 37 °C and different pH conditions (pH 3.0–7.0) for up to 21 days. The color deterioration and loss in antioxidant capacity of HP betacyanins were effectively alleviated as well. In vitro gastrointestinal digestion revealed that the retention rate of HP betacyanin was significantly improved. This was a preliminary study for the application of liposomal technology in the development of a natural colorant based on HP betacyanins with the purpose of improving the storage stability and bioaccessibility of HP betacyanins. The results indicated the feasibility of the attempt. However, liposomal formulation still needs to be optimized to further enhance the stability of the HPBL and better avoid the leakage of encapsulated betacyanins. Moreover, the composition of the HP extracts and their interaction with liposomes also need investigation to better understand the complex system.

## Figures and Tables

**Table 1:** Central Composite Design Matrix and The Response Values for The EE of Betacyanin in HPBL.

No.	A: HP Extract Concentration (g/mL)		B: Lecithin-to-Cholesterol Ratio		C: Lecithin Concentration (g/mL)		EE (%)
	X1	Code X1 *	X2	Code X2 *	X3	Code X3 *	
1	0.125	0	0.000	−α	0.018	0	90.34%
2	0.125	0	5.000	0	0.018	0	88.19%
3	0.125	0	5.000	0	0.006	−α	93.28%
4	0.200	α	5.000	0	0.018	0	61.72%
5	0.080	−1	7.973	1	0.025	1	85.80%
6	0.170	1	2.027	−1	0.011	−1	80.41%
7	0.125	0	5.000	0	0.018	0	89.19%
8	0.080	−1	2.027	−1	0.011	−1	93.75%
9	0.170	1	7.973	1	0.025	1	74.48%
10	0.050	−α	5.000	0	0.018	0	75.84%
11	0.170	1	7.973	1	0.011	−1	84.65%
12	0.125	0	10.000	α	0.018	0	90.24%
13	0.125	0	5.000	0	0.030	α	87.42%
14	0.125	30	5.000	0	0.018	0	89.66%
15	0.081	−1	2.027	−1	0.025	1	92.05%
16	0.080	−1	7.973	1	0.011	−1	88.93%
17	0.170	1	2.027	−1	0.025	1	83.67%

* The code numbers of 0, −1, 1, −α, and α represented the center point, factorial point, factorial point, axial point, and axial point in the central composite design, respectively.

**Table 2:** Analysis of Variance of Regression Model.

Variables	Sum of Squares	df	Mean Square	F-Value	*p*-Value Prob. > F
Model	1015.64	9	112.85	15.98	0.0007
A-HP extract concentration	264.15	1	264.15	37.41	0.0005
B-Lecithin-to-cholesterol ratio	16.67	1	16.67	2.36	0.1683
C-Lecithin concentration	31.06	1	31.06	4.4	0.0742
AB	3.28	1	3.28	0.464	0.5176
AC	1.19	1	1.19	0.1688	0.6935
BC	31.42	1	31.42	4.45	0.0729
A^2^	489.34	1	489.34	69.3	<0.0001
B^2^	11.91	1	11.91	1.69	0.2353
C^2^	12.15	1	12.15	1.72	0.231
Lack of fit	48.3	5	9.66	17.11	0.0561
Residual	49.43	7	7.06		
Pure error	1.13	2	0.5646		
Correlation total	1065.07	16			

## Data Availability

Not applicable.

## Sample Availability

Samples of the compounds are not available from the authors.
